# The combination of intravitreal triamcinolone and phacoemulsification surgery in patients with diabeticfoveal oedema and cataract

**DOI:** 10.1186/1471-2415-5-15

**Published:** 2005-06-22

**Authors:** Maged S Habib, Paul S Cannon, David HW Steel

**Affiliations:** 1 Sunderland Eye Infirmary, Queen Alexandra Road, SR2 9HP, UK

## Abstract

**Background:**

The management of diabetic patients with refractory macular oedema or patients with no adequate pre-operative view to administer laser treatment provide a challenge to the ophthalmologist. We wished to assess the use, safety and effect of intravitreal triamcinolone injection at the time of cataract surgery in patients with diabetic foveal oedema and sight limiting lens opacities.

**Method:**

This was a longitudinal non-randomised prospective pilot study in 18 eyes (12 patients). All patients had visually significant lens opacities and either persistent diabetic foveal oedema unresponsive to laser treatment-group A, or foveal oedema with no adequate pre-operative view for laser treatment- group B. The cataract surgery was carried out under full aseptic technique using a self-sealing temporal incision and a foldable acrylic lens. Intravitreal triamcinolone was given infratemporally pars plana at the completion of the cataract surgery. The patients were reviewed at day 5, 2 weeks, 2 months and then every 3 months as required. The Wilcoxin matched-pairs test was used to assess the significance of the improvement in visual acuity at 2 months.

**Results:**

Twelve patients with a total of 18 eyes were included in the study. There were 10 patients (15 eyes) in group A and 3 patients (3 eyes) in group B. Preoperatively 16 of the 18 eyes had a visual acuity of 6/24 or worse. Postoperatively 83% of patients had completely dry foveae at 2 weeks. Best-corrected visual acuities at two months review ranged from 6/6 to CF with 9 eyes (50%) achieving 6/12 or better (7 eyes (47%) in group A and 2 eyes (67%) in group B). Three eyes had no recorded improvement in visual acuity, but no eyes had deterioration in acuity. The improvement in visual acuity was significant at p = 0.001. There were no significant sight threatening complications.

**Conclusion:**

Intravitreal triamcinolone has been shown to lead to an improvement in macular oedema and visual improvement in diabetic patients not undergoing cataract surgery but has not, to our knowledge, been previously used in a study like this one.

We suggest that intravitreal injection at the time of cataract surgery could be carried out safely with encouraging visual outcomes in patients with diabetic foveal oedema and cataract.

## Background

Diabetes mellitus is the most common predisposing risk factor for cataracts in the developed world with a three to four fold increased prevalence in people under 65 [1]. It is an important cause of reduced vision [2] and it has been estimated that up to 20% of all cataract surgery is performed on diabetic patients [3].

Cataract surgery in patients with diabetic retinopathy is associated with an increased risk of a number of problems including uveitis [4], posterior capsule opacity [5], and anterior capsule phimosis [6]. Some studies have found an increased risk of retinopathy progression and in particular macular oedema exacerbation with cataract surgery [7-12]. Cataracts can also impair the recognition of sight threatening retinopathy and obstruct treatment of maculopathy.

It is thought that the incidence of these complications and the visual outcome is related to the severity of the retinopathy and its activity at the time of surgery as well as glycaemic control [12,13]. Patients with no retinopathy have an excellent prognosis [14,15]. However, patients with macular oedema have a poor visual prognosis [14-21]. Macular oedema presenting de novo immediately following cataract surgery can spontaneously resolve with a good result, and patients with previously successfully treated macular oedema do well with cataract surgery [22]. However persistent macular oedema despite laser or macular oedema either undetected or untreatable prior to surgery does not resolve spontaneously. These patients have a substantially impaired prognosis [14-21].

Although laser photocoagulation can be applied post-operatively it can be difficult to perform immediately following surgery, because of problems such as photophobia, contact lens intolerance, poor mydriasis, intraocular lens deposits and edge effects, posterior capsule opacity or vitreous haemorrhage. Intra-operative pan retinal photocoagulation (PRP) can be applied with an indirect ophthalmoscope, however intra-operative focal laser applied in this way is imprecise. It is recognised that the increased blood ocular barrier breakdown associated with pan retinal photocoagulation can potentially exacerbate macular oedema [23].

Intravitreal triamcinolone has been successfully used to treat patients with refractory diffuse diabetic macular oedema [24-26] and patients with refractory pseudophakic macular oedema [27,28]. It has also been shown to reduce blood ocular barrier break down following PRP [29].

We designed an initial pilot study to assess the ease of use, safety and effect of intravitreal triamcinolone injection at the time of cataract surgery in patients with sight limiting cataract and diabetic foveal macular oedema.

## Methods

We carried out a longitudinal pilot study of 12 patients (18 eyes) undergoing phacoemulsification cataract surgery. Inclusion criteria was visually significant cataract with either:

a) Pre-existing and persistent diabetic foveal oedema unresponsive to laser treatment as per ETDRS recommendations [30] – Group A.

b) Foveal oedema with no adequate view to perform pre-operative laser – Group B.

Exclusion criteria included a known increased intraocular pressure response to steroids and glaucoma. Fully informed consent was taken. Patients were examined immediately prior to surgery by recording the best-corrected visual acuity, intraocular pressure and slit lamp biomicroscopy using a 66 dioptre fundal lens. Wisconsin retinopathy grade [31] was recorded at each visit.

Surgical technique included full asepsis with povidone-iodine washout of conjunctival sac pre-operatively and subconjunctival cefuroxime at the completion of surgery. All surgeries were completed under topical anaesthesia with a self-sealing temporal corneal tunnel and a 6 mm folding acrylic lens (SA60- Alcon).

At the completion of cataract surgery all patients had 4 mgs in 0.1 ml of triamcinolone acetate injected via the inferotemporal pars plana, using a 27-gauge needle. The injection was directed into the inferior vitreous cavity to reduce the incidence of visually disturbing floaters post-operatively and was given inferior to the temporal corneal tunnel to avoid anterior chamber shallowing during scleral perforation of the needle. Patients were seen on day five, two weeks, two months and then three monthly depending on clinical assessment. Best-corrected visual acuities were obtained at two months post-operatively with refraction.

## Results

There were 12 patients with a total of 18 eyes. There were 10 patients (15 eyes) in group A and 3 patients (3 eyes) in group B. There was one patient with one eye in group A and one eye in group B. There were 8 females and 4 males with a mean age of 69 years. Eleven of the twelve patients were type two diabetics of which three were taking insulin. Mean Haemoglobin A1C at the time of surgery was 8.7% (range 7.2–11.4%) and mean duration of diabetes was 9 years (ranges 2–25 years). All of the 11 type two diabetic patients were being treated for hypertension.

Pre-operative visual acuities ranged from 6/12 to hand movements secondary to a combination of cataract and maculopathy, with 16 of the 18 eyes having a visual acuity of 6/24 or worse. Two patients in group B had intra-operative pan retinal photocoagulation (but not focal laser) applied during phacoemulsification surgery. [Table 1]

Best-corrected visual acuities at two months post-operatively ranged from 6/6 to CF with 9 eyes (50%) achieving 6/12 or better (7 eyes (47%) in group A and 2 eyes (67%) in group B). No eyes had deterioration in visual acuity but 3 eyes (patients 4 and 6) had no recorded improvement in acuity despite the fovea being clinically dry in two of these cases (patient 6). [Table 2] The improvement in visual acuity was significant at p = 0.001 using a Wilcoxon matched-pairs test.

Fifteen eyes (83%) had clinically dry foveae at two weeks post surgery – 12 (80%) in group A and all 3 (100%) in group B. Last recorded visual acuities at a mean time of 8.5 months showed a deterioration from best-recorded visual acuity in 5 (27%) patients because of recurrent macular oedema – all in group A. [Table 2]

Six (33%) eyes; all in group A; showed a one-step or more deterioration in retinopathy grade at last follow up (mean follow up 8.5 months, range 4–15 months) compared with pre-operative levels. All cases of progression occurred five or more months post-operatively. [Table 3]

The triamcinolone was quick and easy to give and there were no immediate operative complications from its administration such as haemorrhage, capsule tears or passage of the triamcinolone into the anterior chamber. Post-operatively 4 patients had transiently raised intraocular pressure requiring treatment with ocular hypotensive drops. None of these patients had pre -existing raised intraocular pressure or were known steroid responders. All 4 patients had normal pressures at the first visit but 2 developed raised pressure at two weeks(IOPs 34 and 29 mmHg) and the other 2 by 2 months (IOPs 31 and 24 mmHg). [Table 3] Three patients (patients 2, 4 and 8) were treated successfully with topical beta blockers as a monotherapy and 1 (patient 5) required combination therapy with a topical beta blocker and a topical carbonic anhydrase inhibitor. At 6 months post-operatively all pressures were normal off treatment. Interestingly 2 patients (patients 2 and 4) who had both eyes operated upon, developed high pressure in their second eyes after having had no pressure rise in their first eye.

Three patients complained of visual floaters post-operatively which settled within a few days. The retinal view was unimpeded and accurate clinical examination was possible. There were no cases of endophthalmitis, uveitis, significant capsule phimosis or posterior capsule opacity in the follow-up period. [Table 3]

## Discussion

This pilot study suggests that intravitreal triamcinolone can be given safely and easily at the time of phacoemulsification surgery.

We took care to inject the triamcinolone inferotemporally away from the visual axis and anterior chamber to avoid visually troublesome floaters and transit of triamcinolone into the anterior chamber. The retinal view was unimpaired post-operatively allowing accurate retinal assessment and further laser treatment if needed. Similar to other studies [32,33] we found only 22% of eyes developed increased intraocular pressure and all were treated successfully with topical ocular hypotensive agents with spontaneous improvement with time.

Intravitreal triamcinolone has been shown to lead to an improvement in macular oedema and visual improvement in diabetic patients not undergoing cataract surgery [24-26] but has not; to our knowledge; been previously studied in a series such as this.

Combining cataract surgery with triamcinolone rather than giving triamcinolone before surgery as a separate procedure avoided the potential for progression of lens opacities associated with intraocular steroids [34,35] which could have further interfered with retinopathy assessment. We had no cases of endophthalmitis, which can occur with triamcinolone injections [36,37]. Combining the two procedures reduces the patient's potential risk of endophthalmitis from two separate intraocular episodes to one, whilst at the same time offering improved patient convenience. The technique was simple adding very little time to the procedure and in this series there was no significant ocular morbidity associated with the triamcinolone. We choose to combine the procedure with our standard clear corneal temporal incision phacoemulsification technique. There is debate regarding the possibility of an increased risk of endophthalmitis with temporal clear corneal incisions [38]. However this has not been our experience. We have had no increase in our rate of endophthalmitis over the last five years during which a clear corneal temporal approach has been adopted by all surgeons at our unit. Incidence of endophthalmitis in our unit for 1999 was 0.11% and for 2004 was 0.09%, based on approximately 6000 cases /year [39]. It is possible that other factors such as wound construction and lid draping and preparation are more important than incision position itself [38]. All the cases in this series were done in theatre with full asepsis, topical povidone pre-operatively, careful lid draping and carefully constructed wounds, which were watertight at the close of surgery. These are important factors in avoiding infective complications.

We had no cases of posterior capsule rupture in this series. Triamcinolone has been used to help visualise vitreous during posterior capsule rupture and anterior vitrectomy [40] and anecdotally in cases of phacoemulsification surgery with posterior capsule rupture to reduce the incidence of post operative cystoid macular oedema and postoperative inflammation [41]. Potentially therefore intravitreal triamcinolone administration could be considered even if posterior capsule rupture was to occur although we have no experience of this.

The natural history of patients with foveal oedema at the time of cataract surgery is recognised as being poor and the patients in the study had a number of other features associated with a particularly poor prognosis after cataract surgery- increasing age, female sex, poor glycaemic control with high Haemoglobin A 1C (%) at the time of surgery and moderate to severe background retinopathy changes, have all been associated with a poor prognosis in other studies [12,13,16,18]. All the patients in group A had chronic macular oedema prior to cataract surgery, which had been unresponsive to treatment. Indeed patients such as these with chronic unresponsive macular oedema, particularly if there is only a moderate degree of cataract, are often declined surgery on the basis that the maculopathy would limit the underlying visual acuity, which can also deteriorate with surgery. Despite this approximately 50% of these patients achieved 6/12 vision.

Patients who present with dense cataracts and significant retinopathy, especially maculopathy, which is untreatable pre-operatively because of the lens opacities, pose a difficult clinical scenario. Laser can be performed post-operatively but this can be difficult, for reasons previously stated and surgically induced inflammation. There were three patients in the study in this group – group B. Two of these patients had very severe non proliferative retinopathy, in addition to maculopathy, and were treated with intra-operative PRP which can also exacerbate macular oedema. Despite these difficulties and risk factors for maculopathy exacerbation all three patients had complete macular oedema resolution at the two week examination without any macular laser having been applied at that stage.

Overall fifteen (83%) of the patients had complete resolution of their macular oedema at two weeks follow up. Recurrence of macular oedema occurred in some patients in this study as would be expected from the known short term effect of intravitreal triamcinolone and other studies with intravitreal triamcinolone and diabetic macular oedema. However the triamcinolone clearly prevented the short term exacerbation of macular oedema that can be associated with blood ocular breakdown due to intraocular surgery and PRP [23,29,42]. It seems logical to use a drug, albeit with a known short-lived effect, in this way to potentially improve visual outcome until longer lasting alternatives are produced.

Retinopathy progression occurred during follow up in only six patients (none within 5 months of surgery and 4 out of 6 at more than 10 months following surgery) and it may be that triamcinolone has a role in reducing the deterioration of retinopathy that has been reported following cataract surgery especially in patients with more advanced retinopathy. It may also have a role in the inhibition of retinal neovascularisation [43,44]. Numbers in this uncontrolled pilot study are too limited to draw any definitive conclusions.

Triamcinolone was only injected in those eyes with pre-existing macular oedema at the time of cataract surgery. It was not used in eyes thought to be at risk of developing macular oedema after surgery in those with clinically dry foveae at the time of surgery. The natural history of patients with dry maculae at the time of surgery who develop macular oedema following surgery is relatively good [22] and it was felt that the risks associated with triamcinolone in that group would outweigh the benefits. At present we are not recommending triamcinolone in that group of patients, although this may merit further investigation.

## Conclusion

This pilot study suggests that intravitreal triamcinolone can be given safely and easily at the time of phacoemulsification surgery in patients with visually significant cataract and diabetic foveal macular oedema. Eighty-three percent of the eyes in this study had complete resolution of macular oedema at two weeks post-operatively and none experienced any exacerbation of their maculopathy. Further controlled studies are needed to demonstrate the potential visual advantages and long-term benefit of this treatment approach.

## Competing interests

The author(s) declare that they have no competing interests.

## Authors' contributions

DS conceived of the study, participated in the design of the study and helped to draft the manuscript. MH participated in the design and the coordination of the study. PC participated in the coordination of the study and helped to draft the manuscript.

## Pre-publication history

The pre-publication history for this paper can be accessed here:



## Figures and Tables

**Table 1:** Pre-operative clinical characteristics of patients.

**Group**	**Patient number**	**Age (years)**	**DM type**	**Pre-op PRP**	**Pre-op Va**	**Wisconsin Retinopathy Grade Pre-op**
**A**	1	65	II	No	6/60	Moderate
				No	CF	Moderate
**A**	2	77	II	No	6/60	Moderate
				Yes	CF	LRPDR
**A**	3	73	II	No	6/24	Mild
**A**	4	71	II	No	6/60	Moderate
				No	CF	Severe
**A**	5	67	II	Yes	HM	HRPDR
**A**	6	72	II	No	6/36	Moderate
				No	6/24	Moderate
**A**	7	76	II	No	6/24	Mild
**A**	8	68	II	No	6/12	Mild
				No	6/18	Moderate
**A**	9	71	II	No	6/60	Severe
**A**	10	67	II	No	6/36	Moderate
						
**B**	10			No (intra-op)	HM	Very Severe
**B**	11	69	II	No (intra-op)	HM	Very Severe
**B**	12	49	I	No	6/36	Mild
		**Mean age = 68.75**				

DM = Diabetes Mellitus; I = type one diabetes; II = type two diabetes; Pre-op = pre-operatively; PRP= pan retinal photocoagulation; Va = visual acuity; intra-op = intra-operatively; CF = counting fingers; HM = hand movements; LRPDR = low risk proliferative diabetic retinopathy; HRPDR= high risk proliferative diabetic retinopathy

**Table 2:** The post-operative clinical and visual outcomes of patients.

**Group**	**Patient Number**	**Refracted Va two months post surgery**	**Last Va**	**Macula clinically dry at two weeks**	**Macula clinically dry at last FU**
**A**	1	6/9	6/18	Yes	Yes
		6/9	6/12	Yes	Yes
**A**	2	6/12	6/12	Yes	Yes
		6/36	6/36	No	No
**A**	3	6/18	6/18	Yes	Yes
**A**	4	6/9	6/12	Yes	No
		CF	CF	No	No
**A**	5	6/60	6/60	No	No
**A**	6	6/36	6/36	Yes	No
		6/24	6/36	Yes	No
**A**	7	6/6	6/6	Yes	Yes
**A**	8	6/9	6/9	Yes	Yes
		6/9	6/12	Yes	Yes
**A**	9	6/24	6/24	Yes	No
**A**	10	6/18	6/18	Yes	Yes
					
**B**	10	6/18	6/18	Yes	No
**B**	11	6/12	6/12	Yes	Yes
**B**	12	6/6	6/6	Yes	Yes

Va = visual acuity; FU = follow up; CF = counting fingers

**Table 3:** Post-operative complications and deterioration in retinopathy grade on follow-up.

**Group**	**Patient Number**	**Follow up (months)**	**Raised IOP**	**Stepwise deterioration in retinopathy grade at last FU (ETDRS)**
**A**	1	15		3
		14		1
**A**	2	6		0
		8	Yes: 34 mmHg	0
**A**	3	9		0
**A**	4	10		0
		12	Yes: 29 mm Hg	1
**A**	5	11	Yes: 31 mm Hg	0
**A**	6	10		1
		7		0
**A**	7	12		0
**A**	8	5	Yes: 24 mmHg	1
		5		0
**A**	9	7		1
**A**	10	6		0
				
**B**	10	4		0
**B**	11	8		0
**B**	12	4		0
		**Mean FU = 8.5**		

IOP = intraocular pressure; FU = follow-up; ETDRS = Early Treatment Diabetic Retinopathy Study
